# Rethinking altitude and child growth: evidence from a multi-altitude study in Tibet

**DOI:** 10.3389/fnut.2025.1648246

**Published:** 2025-09-24

**Authors:** Shubin Shao, Xiaowei Ma, Dawa Wangyai, Jian Wang, Agudamu, Yang Zhang

**Affiliations:** ^1^Education Bureau of Chamdo City, Chamdo, China; ^2^Tianjin University of Sport, Tianjin, China; ^3^Laboratory of Physical Health Integration and Health Promotion, Tianjin Sports Institute, Tianjin, China; ^4^Chamdo No. 4 High School, Tibet, China; ^5^College of Physical Education, Qinghai University for Nationalities, Qinghai, China; ^6^Hunan Geely Automobile College, Hunan, China; ^7^Independent Researcher, Windermere, FL, United States

**Keywords:** BMI, high altitude, overweight, socioeconomic disparity, stunting, undernutrition

## Abstract

**Objective:**

High-altitude environments have long been linked to growth deficits in children, often attributed to chronic hypoxia. Yet, emerging evidence from better-resourced highland communities suggests that socioeconomic and infrastructural factors may be equally or more influential. This study evaluated growth and nutritional disparities among Tibetan children across altitudes and assess the continued relevance of the altitude-growth paradigm.

**Methods:**

A repeated cross-sectional study in 2019 and 2024 surveyed 28 schools in Qamdo, Tibet (3200–4,500 m), collecting anthropometric and lung function data from 8,230 children (47.4% boys) aged 6–17 years. Regional growth standards were updated using GAMLSS. Logistic regression examined associations of age, sex, and altitude with stunting, underweight, and overweight, using girls and ~3,200 m as the reference groups.

**Results:**

In 2024, adolescent height approached national norms, with marked gains since 2019. Stunting (12.3%) and underweight (9.2%) remained prevalent. Compared to ~3,200 m, children at ~3,600 m had higher odds of stunting (OR 6.10, 95% CI 4.94–7.58) and underweight (OR 9.03, 95% CI 7.05–11.63), while >4,000 m showed smaller increases (stunting OR 3.43; underweight OR 4.63). Boys had greater odds of stunting (OR 1.16, 95% CI 1.02–1.33) and underweight (OR 1.34, 95% CI 1.15–1.56) than girls. Body mass index rose in 2024 but remained below national averages; body fat was consistently lower.

**Conclusion:**

Non-linear altitude effects and catch-up growth under improved conditions suggest altitude is an indirect risk factor mediated by modifiable socioeconomic constraints, challenging deterministic altitude-growth models and supporting targeted nutrition and infrastructure policies.

## 1 Introduction

Human growth is a sensitive marker of environmental quality and social development, reflecting the cumulative influence of nutrition, disease burden, physical activity, and psychosocial stressors throughout childhood. In low-resource or ecologically challenging environments, these stressors often manifest as growth faltering, most notably in the form of stunting and underweight. Among these environments, high-altitude regions present a unique biological and ecological context where oxygen deprivation, or hypoxia, has long been suspected to interfere with typical growth trajectories (1). Indeed, the altitude-growth paradigm posits that chronic hypoxia constrains oxygen availability for metabolic and cellular processes (2, 3), thereby limiting linear growth in highland populations. Environmental stressors such as low temperatures and elevated ultraviolet radiation may further restrict growth (4). Supporting this view, historical and contemporary data have consistently shown that children living at high altitudes tend to be shorter and lighter than their lowland peers, even after adjusting for potential confounders such as nutrition and income (5). Some scholars therefore have argued that these growth patterns may represent adaptive phenotypes rather than pathological delays (6, 7).

To empirically examine these theoretical claims in the Tibetan context, Ma et al. conducted a field study in 2019 in a county located at approximately 3,650 meters above sea level (hereafter m) (8). The results revealed that children in the region had significantly lower height and weight values compared to the national reference standard. These findings underscored the inadequacy of national benchmarks for high-altitude populations and prompted the development of the first Tibetan child-specific growth reference. While this reference has served as a practical tool for local health assessment, its applicability is limited. It was constructed using data from a single altitude band and does not account for interregional variations in economic development, nutritional access, and healthcare infrastructure—factors that may critically influence child growth outcomes even at similar altitudes.

Since the publication of this regional reference, emerging evidence from other highland areas in Tibet has begun to challenge the deterministic view of the altitude-growth paradigm. In particular, children living in better-resourced communities such as Lhasa (9) have demonstrated growth trajectories that exceed those predicted solely by altitude-based physiological models. These discrepancies suggest that altitude may act more as a contextual modifier than a fixed biological constraint, with growth outcomes influenced by complex interactions between hypoxia and modifiable environmental factors, including school nutrition programs, food security, and primary healthcare access (10–12). As such, it is increasingly necessary to reevaluate the relative contributions of altitude to childhood growth in highland regions.

The purpose of the present study was to assess growth development among children living at different altitudes across the Tibetan Plateau, and to examine how altitude shape undernutrition risk. By analyzing a large, multi-site dataset, we aim to identify spatial disparities in child growth, validate the generalizability of the existing Tibetan growth reference (8), and generate evidence to inform more context-appropriate health monitoring and intervention strategies in high-altitude settings.

## 2 Methods

### 2.1 Participants

This study was designed and organized by the Education Bureau of Chamdo City as part of the “Tibetan Student Physical Fitness and Health Standards” program. All sample locations were pre-determined in the official government implementation document, ensuring geographic coverage across key altitude bands within Qamdo. As the survey was conducted as a government-led census of all students in the selected schools, no *a priori* sample size calculation was performed.

This repeated cross-sectional study combined anthropometric data from a 2019 field survey (Jomda County) and a 2024 survey across eight other regions within Qamdo in Tibet. The surveyed regions included Baxoi (3,200 m), Karub (3,200 m), Qamdo (3,250 m), Jomda (3,650 m), Tongka (4,100 m), Bangda (4,200 m), Jizhong (4,300 m), Yiqing (4,400 m), Guoqing (4,500 m), and Latuo (4,500 m). Altitudes were grouped into three bands (~3,200 m, ~3,600 m, and >4,000 m) to reflect ecologically meaningful variation and support statistical comparisons. Both surveys were conducted in September to coincide with the school term and avoid weather-related disruptions.

All participants were ethnic Kham Tibetan aged 17 years or younger. Children with medical conditions known to affect physical growth were excluded from the study. Written informed consent was obtained from each participant’s legal guardian prior to enrollment. The study protocol received ethical approval from the Ethics Committee of Tianjin University of Sport (Approval No. TJUS 2024–070).

### 2.2 Procedures

Data collection was conducted by trained researchers and included assessments of health indicators and physical fitness performance, with the latter to be reported separately. To ensure measurement reliability, all equipment was calibrated onsite, and a briefing session was held with local teachers to address procedural questions. On the assessment day, students gathered in an indoor area and were instructed to remain seated quietly until individually called for evaluation. Anthropometric measurements were collected first. Height was measured to the nearest 0.1 cm using portable stadiometers, and weight was recorded to the nearest 0.1 kg using calibrated electronic scales, which was used to calculate body mass index (BMI). Female examiners assessed triceps and subscapular skinfold thickness using a Holtain Tanner caliper (Holtain, UK). Three readings were taken at each site, and the mean values were used to estimate body fat percentage, following established protocols (13). Vital capacity was measured using a portable spirometer (WQS8888, Shanghai Wanqing Rlrctron, China), with each child performing three trials; the highest value was retained for analysis.

### 2.3 Statistics

Data analysis was conducted using R, incorporating the packages *gamlss* (version 5.4–22), *ggpubr* (version 0.6.0), and *broom* (version 1.0.8). Statistical significance was defined as a two-sided *p*-value below 0.05. Cases with missing or incomplete data were excluded from the relevant analysis but retained for descriptive statistics when available. No imputation was performed due to the nature of the cross-sectional design.

To validate the Tibetan growth reference developed in 2019, we refitted Generalized Additive Models for Location, Scale, and Shape using 2024 data. First, we selected three distributions that facilitate interpretation—Box–Cox Cole and Green, Box–Cox power exponential, and Box–Cox *t*—in which *μ* represents approximately the median of the distribution. Second, we determined the optimal distribution and degrees of freedom for smoothing based on the smallest Akaike Information Criterion. Third, all models were fitted using default link functions and a local generalized P-splines smoother, with the penalty parameter set to either two, *ln*(n), or square root of *ln*(n), where n is the number of observations. For quick diagnostics, we assessed the goodness of fit for each model using normalized quantile residuals. New curves were generated for height-for-age, weight-for-age, BMI-for-age, and vital capacity-for-age indicators. Comparisons were made with national norms to assess external validity (13–16).

The subanalysis focused on primary school children aged 7 to 12 years—a critical developmental window with sufficient representation across altitude groups—to explore altitude-related differences in stunting risk. The Kruskal–Wallis test was applied, followed by Wilcoxon tests for pairwise comparisons.

Separate binary logistic regression models were fitted to assess associations between demographic variables and the likelihood of being classified as stunted, underweight, or overweight. For each model, the dependent variable was a binary classification outcome (reference = “normal”). Independent variables included age (continuous), sex (reference = “girls”), and altitude band (reference = “~3,200 m”). Stunting was defined according to national guidelines: for children aged 7 to 17 years, the criterion followed the “Standard for Height Level Classification among Children and Adolescents Aged 7–18 Years” (WST 612–2018), while children aged 6 years were assessed using the “Growth Standard for Children Under 7 Years of Age” (WS/T 423–2022). In both cases, stunting was determined as height-for-age and sex falling below two standard deviations from the national mean. Underweight status was classified using the 2009 Chinese weight-for-age growth charts (14), with thresholds set at below the third percentile. BMI categories were based on age- and sex-specific cutoffs outlined in the “Screening for Overweight and Obesity among School-Age Children and Adolescents” (WS/T 586–2018). Participants were categorized as having either normal weight or overweight; no individuals met the criteria for obesity, and therefore the obesity category was excluded from the analysis. For clarity, the complete age- and sex-specific cutoff tables for BMI and stunting are provided in the online data repository (DOI: 10.6084/m9.figshare.29875658.v1).

## 3 Results

### 3.1 Validation of the 2019 Tibetan growth standard

Table 1 summarizes the sample distribution. After excluding participants with missing key variables, a final sample of 8,230 children was retained for analysis, with data collected from 3,792 children (48.7% boys) in 2019 and 4,438 children (46.3% boys) in 2024.

**Table 1:** Distribution of sample by age and altitude.

Age (yrs)	Boys (*n*)			Girls (*n*)			Total (*n*)
	~3,200 m	~3,600 m	>4,000 m	~3,200 m	~3,600 m	>4,000 m	
6	34	0	13	24	0	8	79
7	54	181	81	67	200	65	648
8	100	307	106	86	353	91	1,043
9	102	403	89	84	372	89	1,139
10	84	329	106	98	348	112	1,077
11	109	268	109	105	289	94	974
12	86	163	78	138	177	49	691
13	93	91	8	122	87	11	412
14	112	56	5	138	60	2	373
15	200	25	0	288	32	2	547
16	328	18	0	463	17	0	826
17	159	6	0	246	10	0	421
Total (*n*)	1,461	1847	595	1859	1945	523	8,230

Table 2 presents the 50th percentile values for anthropometric indicators across years and regions. Height values in 2024 showed marked improvement over 2019, particularly from age 12 onward. By age 17, median heights in the 2024 sample approached national norms, raising questions about the continued need for a distinct Tibetan growth reference. Weight values also increased over time but remained below national benchmarks across all ages (14). BMI levels in the 2024 sample were consistently higher than in 2019, reflecting nutritional progress. Nevertheless, both samples remained below national BMI references (15), and body fat percentages in the 2024 sample were substantially lower than national averages (13), particularly among boys, suggesting persistent nutritional deficits in body composition. Vital capacity measurements also revealed significant improvements. At nearly every age, children in the 2024 sample exhibited higher median lung function compared to 2019 data. Despite this gain, values in most age groups remained below national references (16), indicating that functional recovery may lag behind morphological growth. Together, these results suggest secular gains in height, BMI, and lung capacity among Tibetan children since 2019, but also highlight continued undernutrition and developmental gaps relative to national standards.

**Table 2:** The 50th percentile estimates of anthropometric measurements for Tibetan children living at around 3,200 m (2024 sample), around 3,600 m (2019 sample) and above 4,000 m (2024 sample).

Age	Sex	Height (cm)			Weight (kg)			Body mass index (kg/m^2^)			Body fat (%)		Vital capacity (mL)		
		24’	19’	R1	24’	19’	R2	24’	19’	R3	24’	R4	24’	19’	R5
6	Boys	115	114	119	20.1	20.1	21.3	15.2	15.3	·	·	·	800	782	972
	Girls	116	114	118	20.4	19.8	20.4	15.1	15.1	·	·	·	838	715	883
7	Boys	120	118	125	22.4	21.8	24.1	15.5	15.5	16.7	11.2	14.2	912	907	1,257
	Girls	120	118	124	22.0	21.1	22.6	15.2	15.2	16.0	7.66	11.0	876	807	1,101
8	Boys	126	123	131	24.7	23.5	27.3	15.7	15.6	17.4	11.5	15.4	1,117	1,032	1,537
	Girls	125	122	129	23.9	22.6	25.3	15.4	15.3	16.4	7.54	11.6	1,031	911	1,349
9	Boys	132	127	136	27.5	25.4	30.5	16.0	15.8	18.1	12.0	16.0	1,349	1,157	1797
	Girls	131	127	135	26.5	24.7	28.2	15.6	15.5	16.9	8.18	12.6	1,215	1,030	1,594
10	Boys	136	131	141	30.3	27.5	33.7	16.3	16.0	19.0	11.8	16.6	1,498	1,282	2023
	Girls	136	132	141	29.7	27.3	31.8	16.0	15.7	17.7	12.1	18.0	1,355	1,161	1786
11	Boys	141	136	146	32.8	29.7	37.7	16.6	16.2	19.8	12.3	17.4	1,556	1,408	2,236
	Girls	143	137	147	33.9	30.0	36.1	16.6	16.0	18.5	12.7	18.9	1,529	1,294	1923
12	Boys	146	140	152	36.2	32.0	42.5	17.0	16.4	20.4	12.0	16.6	1756	1,533	2,517
	Girls	149	141	153	38.7	32.7	40.8	17.4	16.3	19.3	13.7	19.9	1784	1,417	2056
13	Boys	153	144	160	41.1	34.3	48.1	17.5	16.6	20.8	8.2	12.3	2,330	1,658	2,880
	Girls	154	145	156	43.2	35.6	44.8	18.2	16.8	20.0	12.8	20.8	2039	1,528	2,191
14	Boys	161	148	166	47.4	36.7	53.4	18.2	16.9	21.2	7.7	12.3	2,568	1783	3,234
	Girls	157	148	158	46.9	38.4	47.8	19.0	17.3	20.6	13.8	22.4	2,236	1,633	2,294
15	Boys	166	152	169	52.9	39.2	57.1	18.8	17.1	21.6	8.5	12.6	3,253	1908	3,534
	Girls	158	151	158	49.5	41.0	49.8	19.8	17.8	21.1	15.5	23.8	2,408	1735	2,370
16	Boys	169	157	171	55.0	41.7	59.4	19.2	17.4	22.1	9.8	13.2	3,537	2033	3,768
	Girls	158	153	159	51.0	43.3	50.8	20.4	18.3	21.5	16.7	23.0	2,556	1832	2,417
17	Boys	169	161	171	54.9	44.1	60.7	19.3	17.7	22.6	9.7	13.4	3,575	2,158	3,955
	Girls	158	155	159	51.7	45.5	51.2	20.9	18.8	21.7	17.0	23.0	2,623	1926	2,450

R1 = reference values from the WST 612–2018 and WST 423–2022; R2 = reference values from Li et al. (14); R3 = reference values from Wang et al. (15); R4 = reference values from Ji and Chen (13); R5 = reference values from Li et al. (16).

### 3.2 Influence of altitude on height development

**Figure 1:**
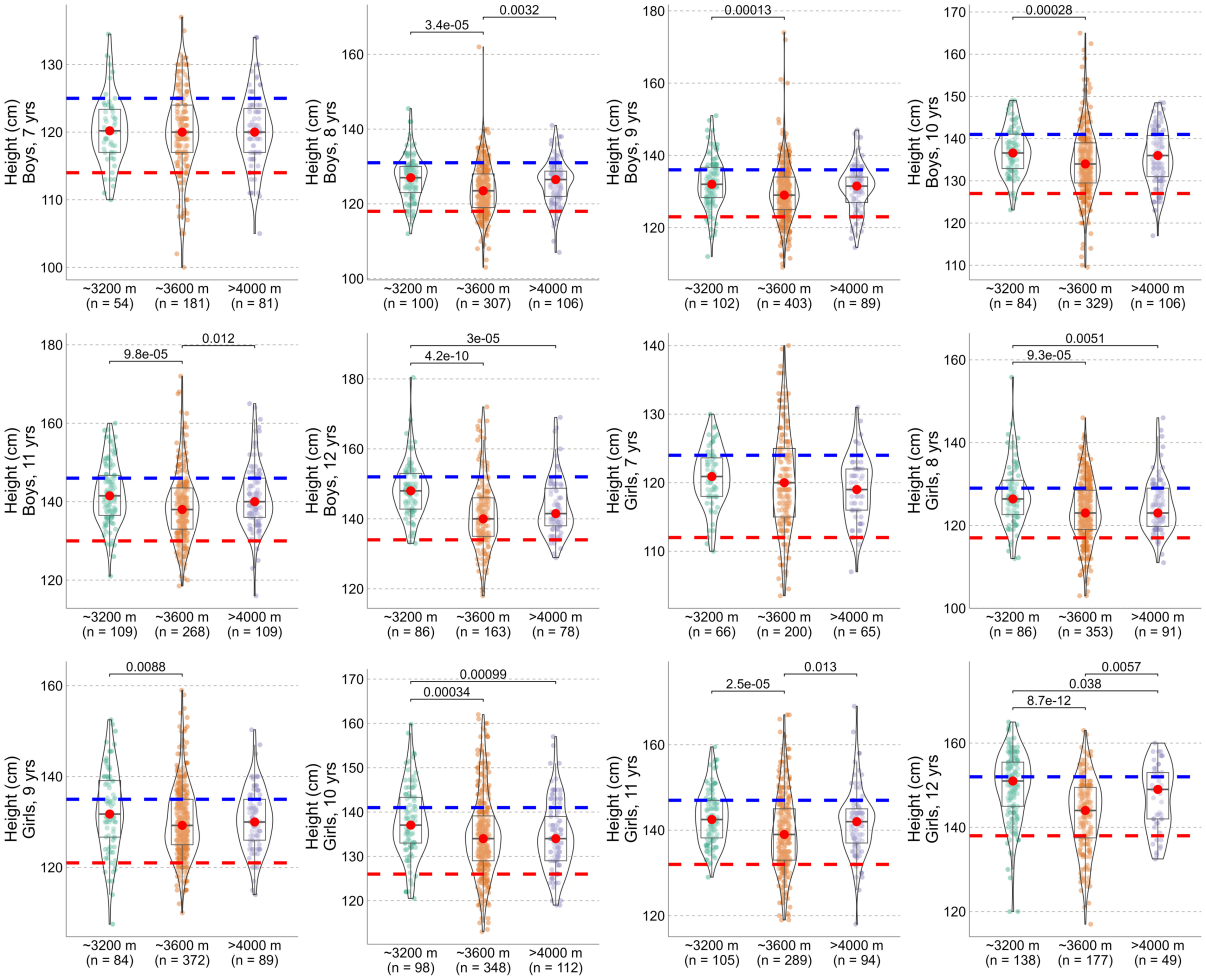
Height development of Tibetan children aged 7–12 years living at different altitudes. Central tendency (median) is indicated by red dots. The dashed red line represents two standard deviations below the national average, while the blue line represents the national median.

Figure 1 displays height distributions by age and altitude band for children aged 7–12. While all altitude groups from the 2024 sample demonstrated age-related growth improvements, no consistent suppressive effect of altitude was observed. In some cases, children living >4,000 m exhibited comparable or even better growth than those at ~3,600 m. These trends challenge the assumption of a direct, dose-dependent relationship between altitude and impaired growth.

### 3.3 Nutritional status

Table 3 presents the binary logistic regression results for each nutritional outcome. In the full 6–17 age sample, older age was significantly associated with higher odds of stunting and underweight, but with reduced odds of overweight. Boys had higher odds of being underweight than girls, while sex was not significantly associated with overweight status. Regarding altitude, children residing at ~3,600 m had significantly elevated odds of both stunting and underweight. The highest altitude group also showed increased odds, although the magnitude of association was generally smaller than that of the ~3,600 m group.

**Table 3:** Nutrition status in Tibetan children.

Nutrition status	Proportion	OR (CI_95%_): Age	OR (CI_95%_): Boys	OR (CI_95%_): ~3,600 m	OR (CI_95%_): >4,000 m
7–12 yrs					
Stunting (*n* = 5,572)	13.4%	1.08 (1.02–1.13)**	0.97 (0.83–1.13)	2.80 (2.19–3.62)***	1.57 (1.16–2.14)**
Underweight (*n* = 5,567)	9.3%	1.32 (1.24–1.41)***	1.18 (0.98–1.41)	3.00 (2.25–4.09)***	1.49 (1.03–2.17)*
Overweight (*n* = 5,567)	8.7%	0.88 (0.83–0.94)***	1.36 (1.13–1.65)**	0.37 (0.30–0.45)***	0.32 (0.23–0.42)***
6–17 yrs					
Stunting (*n* = 8,230)	12.3%	1.09 (1.05–1.12)***	1.16 (1.02–1.33)*	6.10 (4.94–7.58)***	3.43 (2.60–4.52)***
Underweight (*n* = 8,224)	9.2%	1.23 (1.19–1.28)***	1.34 (1.15–1.56)***	9.03 (7.05–11.63)***	4.63 (3.32–6.45)***
Overweight (*n* = 8,224)	9.1%	0.91 (0.89–0.94)***	1.10 (0.95–1.28)	0.37 (0.31–0.45)***	0.35 (0.27–0.46)***

OR, odds ratio.

*p < 0.05; **p < 0.01; ***p < 0.001.

In the subanalysis of children aged 7–12 years, the patterns were broadly consistent. Stunting and underweight were again most pronounced in the ~3,600 m group, while overweight was least prevalent in children living at the highest altitudes. These findings suggest that children living at ~3,600 m face greater nutritional risks than those at even higher altitudes (>4,000 m), further challenging the assumption that growth impairment increases linearly with altitude.

## 4 Discussion

This study offers an updated assessment of child growth and nutritional status across multiple altitude bands in Tibet, providing evidence that challenges the long-held assumption of a linear, altitude-determined growth impairment model. The present findings provide three key insights: height development in aggregate is approaching national norms; undernutrition and body composition disparities remain prevalent, yet multifactorial; and altitude may function more as a proxy for modifiable socioeconomic conditions rather than a direct cause of growth faltering.

Contrary to conventional views that high altitude inherently suppresses growth, the 2024 data show that 17-year-old Tibetan adolescents, across all altitude zones, attained height levels nearly on par with national standards. This represents a notable improvement from the previous 2019 field study and calls into question the deterministic framing of altitude-induced growth limitation. Although hypoxia and reduced oxygen saturation can negatively influence early growth trajectories (2, 3), the observed convergence in adolescent height implies that compensatory catch-up growth is feasible under improved environmental and social conditions.

This improvement aligns with major policy reforms launched nationally and regionally, including China’s National Nutrition Program (2017–2030) (17) and a series of targeted interventions in the Tibet Autonomous Region since 2020. Key among these are the relocation of high school education to lower-altitude areas—notably the city of Qamdo (~3,250 m)—and the implementation of the “three guarantees” policy, which provides students with free meals, accommodation, and tuition in school. Infrastructure expansion, such as the G318 highway, has also enhanced food distribution and access to services in previously isolated communities. Furthermore, the introduction of modern agricultural innovations, like soilless vegetable farming by Shandong Shouguang, has dramatically improved the availability of fresh produce. These collective advances have particularly benefited urban centers like Lhasa, where nutritional conditions increasingly resemble those of inland provinces. Taken together, these developments suggest that altitude itself may not be the root cause of growth restriction. Rather, it acts indirectly—amplifying remoteness and structural deprivation—a distinction that underscores the potential for modifiable, policy-driven improvements in highland child health.

Nonetheless, the results highlight ongoing concerns regarding undernutrition (18). The prevalence of stunting, underweight, low BMI, and reduced body fat remains high, especially among children residing in mid-altitude (~3,600 m) areas. Interestingly, the risk of stunting was greater at ~3,600 m than at >4,000 m, a non-linear pattern inconsistent with altitude as a simplistic growth suppression burden. Instead, it points to complex interactions between geography, food access, and economic opportunity. Logistic regression analyses confirmed that both sex and altitude are associated with nutritional risk, but these associations are mediated by contextual factors. Contextual socioeconomic improvements—rather than altitude per se—hold the key to closing Tibet’s growth deficit. Continued surveillance using regional growth standards and disaggregated altitude-stratified analyses will be crucial in refining local intervention strategies.

We also observed a low but non-negligible risk of overweight in the 2024 cohort. Although undernutrition remains the dominant concern, this emerging trend of overweight highlights a potential nutritional transition that warrants attention. Public health strategies must strike a balance—addressing caloric sufficiency while preventing excess—particularly as socioeconomic conditions improve.

A key strength of this study is the inclusion of a large and geographically representative sample across ecologically meaningful altitude bands. According to government data from December 2023, 133,784 students were enrolled in Qamdo and its surrounding prefectures, and this study covers approximately 6.2% of this population. In the meantime, as a cross-sectional analysis, the study cannot confirm causality. Although secular improvements in height align temporally with policy changes, longitudinal or quasi-experimental studies—such as difference-in-differences designs—are required to establish direct impact. Catch-up growth observed in this cohort underscores that strategic, long-term interventions—rather than temporary or localized efforts—are essential to overcoming the compounded disadvantages associated with geographic remoteness.

In conclusion, this return to Tibet calls for a critical rethinking of the altitude-growth paradigm. While traditional theories have emphasized direct physiological limitations imposed by high altitude, this latest dataset suggests a more nuanced reality: children can achieve normative height growth trajectories when supported by targeted policies and infrastructure improvements. Altitude may serve as a signal of vulnerability, but not an immutable constraint. Simultaneously, the persistence of undernutrition alongside emerging overweight trends underscores the complexity of child nutrition in transitioning regions. Routine monitoring across all Tibetan schools will be essential to track progress and guide resource allocation. Ultimately, effective interventions should prioritize breaking the link between geography and nutritional disadvantage through strategic investments in education, food systems, and public health.

## Data Availability

The raw data supporting the conclusions of this article will be made available by the authors, without undue reservation.

## References

[1] Niermeyer S Andrade Mollinedo P Huicho L. Child health and living at high altitude. Arch Dis Child. (2009) 94:806–11. doi: 10.1136/adc.2008.141838, PMID: 19066173

[2] Beall CM. Detecting natural selection in high-altitude human populations. Respir Physiol Neurobiol. (2007) 158:161–71. doi: 10.1016/j.resp.2007.05.013, PMID: 17644049

[3] Provot S Schipani E. Fetal growth plate. Ann N Y Acad Sci. (2007) 1117:26–39. doi: 10.1196/annals.1402.076 18056035

[4] de Meer K Heymans HSA Zijlstra WG. Physical adaptation of children to life at high altitude. Eur J Pediatr. (1995) 154:263–72. doi: 10.1007/BF01957359, PMID: 7607274

[5] Dang S Yan H Yamamoto S. High altitude and early childhood growth retardation: new evidence from Tibet. Eur J Clin Nutr. (2008) 62:342–8. doi: 10.1038/sj.ejcn.1602711, PMID: 17342161

[6] Beall CM. Two routes to functional adaptation: Tibetan and Andean high-altitude natives. Proc Natl Acad Sci. (2007) 104:8655–60. doi: 10.1073/pnas.0701985104, PMID: 17494744 PMC1876443

[7] Julian CG Wilson MJ Moore LG. Evolutionary adaptation to high altitude: a view from in utero. Am J Hum Biol. (2009) 21:614–22. doi: 10.1002/ajhb.20900, PMID: 19367578 PMC2850611

[8] Ma X Mao Y Wang J Wang X. Anthropometric indices, body function, and physical fitness reference values for Tibetan ethnic children aged 6–17 residing at 3,650 meters above sea level. Front Nutr. (2022) 9:6470. doi: 10.3389/fnut.2022.1036470 PMC9615562 36313099

[9] Dong H Deji Q-z Chun H Awang D-z. Dynamic analysis of Tibetan student's body morphologic index from 2000 to 2014 in Lhasa area. Chin J Child Health Care. (2017) 25:556–60. doi: 10.11852/zgetbjzz2017-25-06-05

[10] Katuli S Natto ZS Beeson L Cordero-MacIntyre ZR. Nutritional status of highland and lowland children in Ecuador. J Trop Pediatr. (2012) 59:3–9. doi: 10.1093/tropej/fms032, PMID: 22752465

[11] Harris NS Crawford PB Yangzom Y Pinzo L Gyaltsen P Hudes M. Nutritional and health status of Tibetan children living at high altitudes. N Engl J Med. (2001) 344:341–7. doi: 10.1056/NEJM200102013440504, PMID: 11172165

[12] Baye K Hirvonen K. Evaluation of linear growth at higher altitudes. JAMA Pediatr. (2020) 174:977–84. doi: 10.1001/jamapediatrics.2020.2386, PMID: 32832998 PMC7445632

[13] Ji C Chen T. Establishment of reference curves for triceps and subscapular skinfold thickness in Chinese children and adolescents. Chin J Sch Health. (2013) 34:779–84. doi: 10.16835/j.cnki.1000-9817.2013.07.004

[14] Li H Ji C Zong X Zhang Y. Height and weight standardized growth charts for Chinese children and adolescents aged 0 to 18 years. Chin J Pediatr. (2009) 47:487–92. doi: 10.3760/cma.j.issn.0578-1310.2009.07.003 19951507

[15] Wang X Zhang F Zhang X Yang J. A study on BMI percentile reference values and curves of children and adolescents aged 7 to 17 in Jiangsu Province. J Nanjing Med Univ. (2022) 42:1627–31. doi: 10.7655/NYDXBNS20221121

[16] Li F Wang T Yang Y. Vital capacity growth curve of primary and secondary school students aged 6-18 in Linzi district of Zibo city in 2017. J Prev Med Inf. (2021) 37:486–91.

[17] Cai L Hu X Liu S Wang L Wang X Tu H. China is implementing the national nutrition plan of action. Front Nutr. (2022) 9:983484. doi: 10.3389/fnut.2022.983484, PMID: 36071936 PMC9441738

[18] Li X Li Y Xing X Liu Y Zhou Z Liu S. Urban–rural disparities in the association between long-term exposure to high altitude and malnutrition among children under 5 years old: evidence from a cross-sectional study in Tibet. Public Health Nutr. (2023) 26:844–53. doi: 10.1017/S1368980022001999, PMID: 36098091 PMC10131156

